# Eculizumab treatment in paediatric patients diagnosed with aHUS after haematopoietic stem cell transplantation: a HSCT-TMA case series from Japanese aHUS post-marketing surveillance

**DOI:** 10.1038/s41409-023-02161-7

**Published:** 2023-12-15

**Authors:** Shuichi Ito, Atsuro Saito, Ayako Sakurai, Kenichiro Watanabe, Shuhei Karakawa, Takako Miyamura, Tomoko Yokosuka, Hideaki Ueki, Hiroaki Goto, Hiroshi Yagasaki, Mariko Kinoshita, Michio Ozeki, Norifumi Yokoyama, Hirofumi Teranishi

**Affiliations:** 1 Department of Pediatrics, Graduate School of Medicine, Yokohama City University, 3-9 Fukuura, Kanazawa-ku, Yokohama, Kanagawa 236-0004 Japan; 2 Department of Hematology and Oncology, Children’s Cancer Center, Kobe Children’s Hospital, 1-6-7 Minatojimaminamimachi, Chuo-ku, Kobe, Hyogo 650-0047 Japan; 3 Department of Pediatrics, Japanese Red Cross Narita Hospital, Iida-cho, Narita, Chiba, 286-8523 Japan; 4 Department of Hematology and Oncology, Shizuoka Children’s Hospital, 860 Urushiyama, Aoi-ku, Shizuoka, 420-8660 Japan; 5 Department of Pediatrics, Hiroshima University Hospital, 1-2-3 Kasumi, Minami-ku, Hiroshima, 734-8551 Japan; 6 Department of Pediatrics, Osaka University Graduate School of Medicine, 2-15 Yamadaoka Suita-shi, Osaka, 565-0871 Japan; 7 Division of Hematology/Oncology, Kanagawa Children’s Medical Center, 2-138-4 Mutsukawa, Minami-ku, Yokohama, Kanagawa 232-8555 Japan; 8 Pediatrics, Nihon University Itabashi hospital, 30–1 Ohyaguchi-kamicho, Itabashi-ku, Tokyo, 173– 8610 Japan; 9 Division of Pediatrics, Faculty of Medicine, University of Miyazaki, 5200 Kihara, Kiyotake-cho, Miyazaki, 889-1692 Japan; 10 Department of Pediatrics, Graduate School of Medicine, Gifu University, 1-1 Yanagido, Gifu, 501-1194 Japan; 11 Department of Pediatrics, Gifu Municipal Hospital, 7-1 Kashima-cho, Gifu, Gifu, 500-8513 Japan; 12 Alexion Pharma GK, 3-1-1 Shibaura Minato-ku, Tokyo, 108-0023 Japan

**Keywords:** Paediatrics, Haematological diseases, Medical research, Diagnosis, Molecularly targeted therapy

## Abstract

Haematopoietic stem-cell transplantation (HSCT)-associated thrombotic microangiopathy (HSCT-TMA) is a serious complication with high mortality. Accumulating evidence suggests that complement dysregulation is potentially involved in the development of HSCT-TMA. We retrospectively analysed the clinical characteristics and outcomes of thirteen paediatric patients who were diagnosed with atypical haemolytic uremic syndrome and treated with eculizumab to manage HSCT-TMA during post-marketing surveillance in Japan. The median time from HSCT to TMA was 31 days (Interquartile range, IQR;21–58) and the median doses of eculizumab was three (IQR;2–5). Seven patients (54%) were alive at the last follow-up while six died due to complications related to HSCT. Six of seven survivors initiated eculizumab after insufficient response to plasma therapy. Following eculizumab treatment, median platelet counts and LDH levels in all survivors significantly improved and renal function improved in 4/7 patients. All survivors possessed potential risk factors of complement overactivation. During the follow-up period after eculizumab discontinuation (median;111.5 days, IQR;95–555), no TMA recurrence was observed. In this analysis, eculizumab showed benefit in over half of this paediatric patient population. Ongoing clinical studies are expected to optimize the treatment regimen of terminal complement pathway inhibitor, and it may become a therapeutic option for paediatric HSCT-TMA in the future.

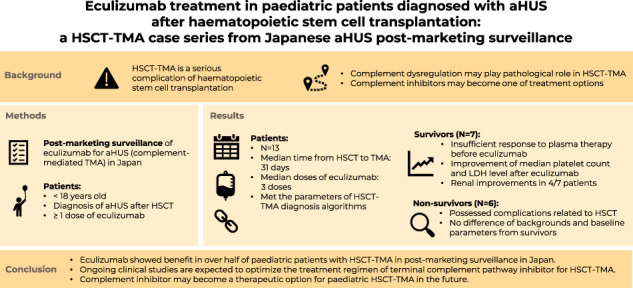

## Introduction

Thrombotic microangiopathy (TMA) is a rare but serious complication that can occur after haematopoietic stem cell transplantation (HSCT) [1]. Although overall survival rates after HSCT have continued to increase due to advances of pre-transplant conditioning regimens and post-transplantation management [2], the treatment strategy of HSCT-TMA remains to be established. The estimated frequency of HSCT-TMA varies in literature ranging from 6–76% following allogeneic HSCT and 0–27% following autologous HSCT [1, 3]. HSCT-TMA involves significant endothelial injury, particularly in the kidney, and is usually fatal unless diagnosed and treated promptly [1, 4]. The survival rate varies among previous reports ranging from 0–78% [3, 5]. Several risk factors for HSCT-TMA have been identified, including graft-versus-host disease (GVHD), medications used for GVHD prophylaxis, donor type, and age [1, 6–8].

A recent report proposed harmonizing definitions for HSCT-TMA diagnostic criteria refining previously proposed criteria [9], but further research is needed to reach a definitive consensus. Although the presence of schistocytes, thrombocytopenia, and elevated lactate dehydrogenase (LDH) levels are the main factors listed in previously proposed criteria for HSCT-TMA, other factors such as renal dysfunction, the result of Coombs test, hypertension, sC5b-9, and neurologic dysfunction differ among the criteria [1, 3, 6, 10–12]. In addition, transfusion due to insufficient stem cell transplant engraftment makes it difficult to diagnose HSCT-TMA because it affects haemoglobin (Hb) level and platelet count, which are indicators for TMA. HSCT-TMA is reported to occur early after HSCT, with 92.3% cases within 100 days of transplant and at a median time of 32 days [13]. Difficulty in reliably diagnosing HSCT-TMA may contribute to the varying percentage of patients reported with HSCT-TMA as well as reported outcomes [1, 3].

Atypical haemolytic uraemic syndrome (aHUS) is a TMA caused by defective complement regulation, and is clinically diagnosed by excluding Shiga-like toxin-producing *E. coli* HUS (STEC-HUS), thrombotic thrombocytopenic purpura (TTP), and secondary TMA. Common characteristics of these diseases include hemolytic anemia, thrombocytopenia, and organ damage caused by thrombosis [1, 3, 6, 10]. Eculizumab is a recombinant humanized monoclonal antibody that binds to and blocks cleavage of C5 to C5a and C5b, thereby inhibiting the terminal complement pathway.

Clinical characteristics of HSCT-TMA such as elevated LDH, decreased haemoglobin (Hb), and kidney dysfunction are similar to that of aHUS. Previous studies have showed elevated plasma C3b, Ba and sC5b-9 levels in patients with HSCT-TMA, which has been thought to be indicative of complement dysregulation [6, 7, 14, 15]. Furthermore, some patients with HSCT-TMA could be predisposed due to presence of complement gene variants [16]. Thus, eculizumab has been considered as a potential treatment option; [17, 18] published case series provide valuable evidence supporting its use for treating paediatric patients’ TMA following HSCT [19–23]. Some of these cases showed haematological responses after initiating eculizumab, but others experienced long-term sequelae or died, presumably because of severe organ damages. No complement inhibitor is approved for HSCT-TMA; therefore, to optimize treatment with complement inhibitor, several clinical trials for HSCT-TMA are ongoing [9].

Eculizumab was approved for the treatment of aHUS in September 2013 in Japan and has been prescribed to patients clinically diagnosed with aHUS [24]. In Japan, all patients clinically diagnosed with aHUS and treated with eculizumab from September 2013 to January 2018 were enrolled in a post-marketing surveillance (PMS). The previous analyses of PMS have demonstrated the safety, tolerability, and effectiveness of eculizumab in a real-world setting in patients with aHUS who did not have a complication including, but not limited to, a history of HSCT, autoimmune disease, and malignant tumour [25–27]. In these analyses, patients who had complications were excluded because it is difficult to distinguish between outcomes from complications and those from aHUS. In the PMS, patients with HSCT-TMA, who were clinically diagnosed with aHUS as an indication for eculizumab treatment, were also enrolled. All of them were excluded in previous analyses; therefore, we focus on this sub-cohort specifically.

## Materials and methods

The design of the PMS, including patient eligibility, data collection, and outcomes, is described in previous reports [26, 27]. This PMS mandated by the Japanese health authority was conducted in accordance with Good Post-Marketing Study Practice (Ministry of Health, Labour and Welfare, Ministerial Ordinance No. 171 of 2004). Ethical approval by an institutional review board and informed consent from individual patients are non-mandatory for PMS. The attending physician provided consent for the use of anonymized data in this report.

Patients were registered in the PMS if they were clinically diagnosed with aHUS and treated with ≥1 dose of eculizumab [26]. The diagnosis of aHUS was based on the latest Japanese clinical guides at the time of diagnosis; these criteria are the presence of TMA symptoms, schistocytes, and relevant exclusion criteria [28–30]. In this report, we focused on paediatric patients (<18 years old) who developed TMA after HSCT. Thirteen patients analysed here were clinically diagnosed with aHUS and treated with eculizumab to manage HSCT-TMA. To examine if they meet the diagnostic criteria of HSCT-TMA, their clinical parameters were retrospectively applied to the items of harmonized diagnosis algorithm recently proposed by Schoettler, et al. [9].

The following data were recorded in the PMS: patient characteristics and medical history, duration of follow-up, number of eculizumab doses, clinical outcomes, laboratory data, response to eculizumab, and safety (adverse events). Data were collected between September 2013 and July 2018, and the database was locked in August 2021; patient outcomes were recorded at the last follow-up prior to database lock. Additional data regarding HSCT related items were collected independently of PMS based on physicians’ consent. Treatment with eculizumab were based on the approved dose for aHUS [24]. Actual dose and administration intervals could be determined by the attending physician.

All data were analysed descriptively as the number and/or percent of patients, or median and interquartile range (IQR) for continuous variables, including changes throughout the observation period, which is defined as from TMA onset to the last follow-up. The timing of TMA onset recorded in the PMS was determined by the attending physician. TMA-related laboratory data were compared between survivors and non-survivors using Wilcoxon’s rank sum test for which two-sided *P*-values of <0.05 were considered statistically significant. The Kaplan–Meier method was used to plot survival and estimate the survival rate at 180 days after initiating eculizumab. SAS version 9.1.3 or later (SAS Institute, Cary, NC, USA) was used for statistical analysis. Estimated glomerular filtration rate (eGFR) was calculated using the formulas specified for Japanese patients aged 2 years through 18 years [31].

## Results

### Patient characteristics

**Fig. 1:**
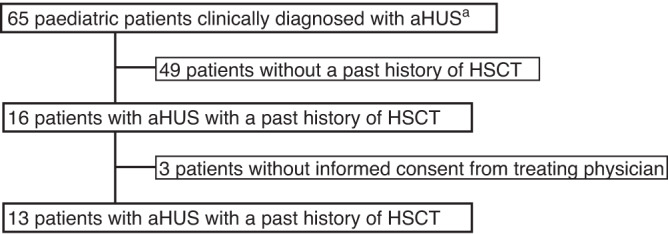
Patient disposition. ^a^Exclude patients with missing records, with diagnosis of STEC-HUS, and without evidence of TMA.

Among 65 paediatric patients who were clinically diagnosed with aHUS and were registered in the PMS,16 patients underwent HSCT before the onset of TMA and thirteen of them were included in this analysis (Fig. 1). Items used to diagnose patients with aHUS and the status of each of the 13 patients are shown in Table S1. Median age for the most recent HSCT was 2 years old (IQR 1–10) (Table 1). Primary diseases included haematological cancers in six patients (acute lymphatic leukaemia, acute myeloid leukaemia, and malignant lymphoma), neuroblastoma in four patients, and familial haemophagocytic lymphohistiocytosis (FHL) in three patients (Table 1).

**Table 1:** Patient characteristics and HSCT treatment regimen.

Pt No	Sex	Age at HSCT, y	Primary disease	Donor type	Allo HSCT			Stem cell source	Conditioning regimen
					Related /Unrelated	HLA match	CI treatment after HSCT (GVHD prophylaxis)		
1^a^	F	2	Neuroblastoma	Allo	Related	Mismatched	Yes	BMT	TBI, Flu, CY
2^a^	M	6	AML	Allo	Related	Mismatched	Yes	BMT	BUS
3	M	0	ALL	Allo	Unrelated	Mismatched	Yes	CBSCT	BUS
4^a^	M	9	Neuroblastoma	Allo	Unrelated	Mismatched	Yes	CBSCT	Flu, L-PAM
5^b,c^	M	0	FHL	Allo	Unrelated	Mismatched	Yes	CBSCT	TBI
6^a,^^b^	M	1	FHL	Allo	Unrelated	Mismatched	Yes	BMT	TBI
7^d^	F	2	Neuroblastoma	Auto	-	-	-	PBSCT	L-PAM, VP-16, CBDCA
8^e^	M	2	Neuroblastoma	Auto	-	-	-	PBSCT	L-PAM, VP-16, CBDCA
9	M	14	ALL	Allo	Related	Mismatched	Yes	BMT	TBI
10^a^	M	17	ALL	Allo	Unrelated	Fully Matched	Yes	BMT	TBI, VP-16, CY
11^a^	M	9	ALL	Allo	Unrelated	Mismatched	Yes	BMT	TBI, L-PAM
12	F	0	FHL	Allo	Unrelated	Mismatched	Yes	CBSCT	TBI
13^f^	M	13	Malignant lymphoma	Auto	-	-	-	BMT	TBI, L-PAM

*ALL* acute lymphocytic leukaemia, *Allo* allogeneic, *AML* acute myeloid leukaemia, *Auto* autologous, *BMT* bone marrow transplantation, *CBSCT* cord blood stem cell transplantation, *CI* calcineurin inhibitor, *F* female, *FHL* familial haemophagocytic lymphohistiocytosis, *GVHD* graft-versus-host disease, *BUS* busulfan, *Flu* fludarabine, *CBDCA* Carboplatin, *CY* Cyclophosphamide, *HLA* human leukocyte antigen, *HSCT* haematopoietic stem cell transplantation, *M* male, *PBSCT* peripheral blood stem cell transplantation, *Pt* patient, *VOD* venoocclusive disease, *VP-16* etoposide, *L-PAM* melphalan, *TBI* total body irradiation.

^a^non-survivors.

^b^These two patients are related.

^c, d,e,f^These patient cases were reported previously [36–39].

### Potential risk factors of HSCT-TMA

Ten out of thirteen patients underwent allogeneic HSCT, and bone marrow was the predominant source of stem cells. Nine out of ten allogeneic HSCT were human leukocyte antigen (HLA) mismatched. All ten patients who underwent allogeneic HSCT received a calcineurin inhibitor as GVHD prophylaxis prior to TMA (Table 1). The median time from HSCT to TMA onset was 31 days (IQR 21–58) (Table 2). Primary disease was classified as ongoing in nine out of thirteen patients at the onset of TMA (Table 2). Eleven out of thirteen patients had complications which might have caused complement overactivation including: seven with coexisting GVHD, three with coexisting veno-occlusive disease (VOD), and eight with a coexisting infection at the onset of TMA (Table 2). Regarding risk factors of aHUS, two patients experienced a previous episode of TMA, and three patients had a family history of TMA. All three patients with a family history of TMA had complement-related gene mutations or were positive for anti-CFH antibodies (Table 2).

**Table 2:** Patient characteristics from haematopoietic stem cell transplantation to thrombotic microangiopathy onset.

Pt No	From HSCT to TMA (days)	Complications at TMA onset			Primary disease condition at TMA onset	History of TMA	Family TMA history	Complement-related factors
		GVHD	VOD	Infection				
1^a^	31	None	None	Fungal infection, CMV pneumonia	Ongoing	0	n/a	n/a
2^a^	32	Ongoing	Ongoing	FN	Ongoing	1	No	n/a
3	58	None	None	None	Ongoing	0	No	n/a
4^a^	26	Ongoing	None	None	Ongoing	0	No	n/a
5	19	Ongoing	Remission	FN	Ongoing	0	Yes	Anti-CFH antibody,
6^a^	21	Ongoing	Remission	None	Ongoing	0	Yes	Anti-CFH antibody,
7	20	-	None	FN	Ongoing	0	Yes	Het *CFHR3*-*CFHR1* del
8	21	-	None	FN	Ongoing	0	No	n/a
9	62	Ongoing	Ongoing	None	Ongoing	0	No	n/d
10^a^	215	Ongoing	Ongoing	pulmonary aspergillosis	None	0	No	n/a
11^a^	323	Ongoing	None	CMV enterocolitis	None	1	No	n/d
12	18	None	None	None	None	0	No	*FCN3* c.465 G > C
13	52	-	None	FN	None	0	No	n/a
Median (IQR), days	31 (21–58)							

*CFH* complement factor H, *CFHR* complement factor H receptor, *CMV* cytomegalovirus, *def* deficiency, *del* deletion, *EB* Epstein–Barr, *ECZ* eculizumab, *FCN3* ficolin 3, *het* heterozygous, *GVHD* graft-versus-host disease, *HSCT* haematopoietic stem cell transplantation, *mut* mutation, *n/a* not available, *n/d* not detected, *PE* plasma exchange, *PI* plasma infusion, *Pt* patient, *TMA* thrombotic microangiopathy, *FN* febrile neutropenia

^a^non-survivors.

### Clinical outcomes

**Fig. 2:**
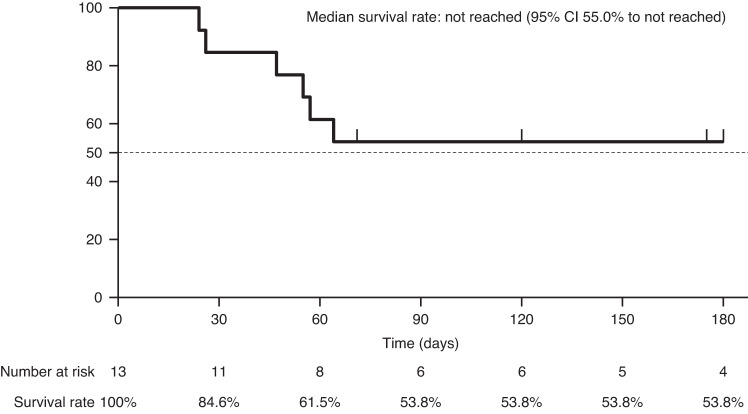
Overall survival rate after initiating eculzumab.

All patients received some form of treatment to manage TMA before eculizumab treatment (Tables 3, S2, and S3). The median time from TMA onset to initiation of eculizumab was 16 days (IQR 8–26). Patient received a median of 3 eculizumab doses (IQR 2–5) (Table 3) with dosing regimen in line with the prescribing information for aHUS [24]. Seven out of thirteen patients survived throughout the observation period (median 172 days, IQR 66–396 days) (Table 3). The Kaplan–Meier estimate for survival rate at 180 days after HSCT-TMA was 53.8% (Fig. 2). Of eight patients who received eculizumab treatment after failure of plasma therapy, six patients survived. Three out of seven survivors received less than 4 eculizumab doses (Table 3).

**Table 3:** Eculizumab treatment and its outcomes.

Pt No	From TMA onset to ECZ initiation(days)	No. of ECZ doses	Prevention of meningococcal infection	PE/PI duration before ECZ initiation (days)	Outcome	Time from TMA onset to last observation/ death (days)	Recurrence of TMA after ECZ discontinuation	Reasons to discontinue ECZ
1^a^	6	2	None^b^	2	Deceased	29	-	Death
2^a^	20	3	Antibiotics	1	Deceased	66	-	Death
3	8	2	None	5	Alive	78	None	Recovered from serious condition^c^
4^a^	3	5	Antibiotics	0	Deceased	57	-	Death
5	26	64	Antibiotics	14	Alive	1557	None(ECZ ongoing)	-
6^a^	126	4	Antibiotics	0	Deceased	182	-	Death
7	8	4	None	4	Alive	182	None	Recovered from serious condition^c^
8	8	3	Antibiotics	3	Alive	776	None	Recovered from serious condition^c^
9	6	1	Antibiotics	6	Alive	742	None	Insufficient response of platelet count^c^
10^a^	16	2	None^b^	0	Deceased	41	-	Insufficient response of platelet count^c^
11^a^	62	5	Antibiotics/Vaccination	0	Deceased	125	-	Death
12	19	10	Antibiotics	0	Alive	396	None	Recovered from serious condition^c^
13	53	3	Vaccination	12	Alive	172	None	Recovered from serious condition^c^
Median (IQR), days	16 (8–26)	3 (2–5)		2 (0–5)	-	172 (66–396)	-	

*AE* adverse event, *CFH* complement factor H, *ECZ* eculizumab, *LDH* lactate dehydrogenase, *PLT* platelet, *Pt* patient, *TMA* thrombotic microangiopathy, *WBC* white blood cell.

^a^non-survivors.

^b^Antibiotics were treated for other coexisting infectious disease.

^c^Treating physician’s comment in PMS.

Eighteen serious adverse events (SAEs) during eculizumab treatment were recorded in nine patients, among which six patients died due to SAEs such as multiple organ dysfunction, adenovirus infection, TMA, cerebral haemorrhage, pseudomonas sepsis, and pulmonary alveolar haemorrhage (Table S4). None of the SAEs leading to death were considered to be related to eculizumab treatment by treating physicians.

### Responsiveness to eculizumab in surviving patients

**Fig. 3:**
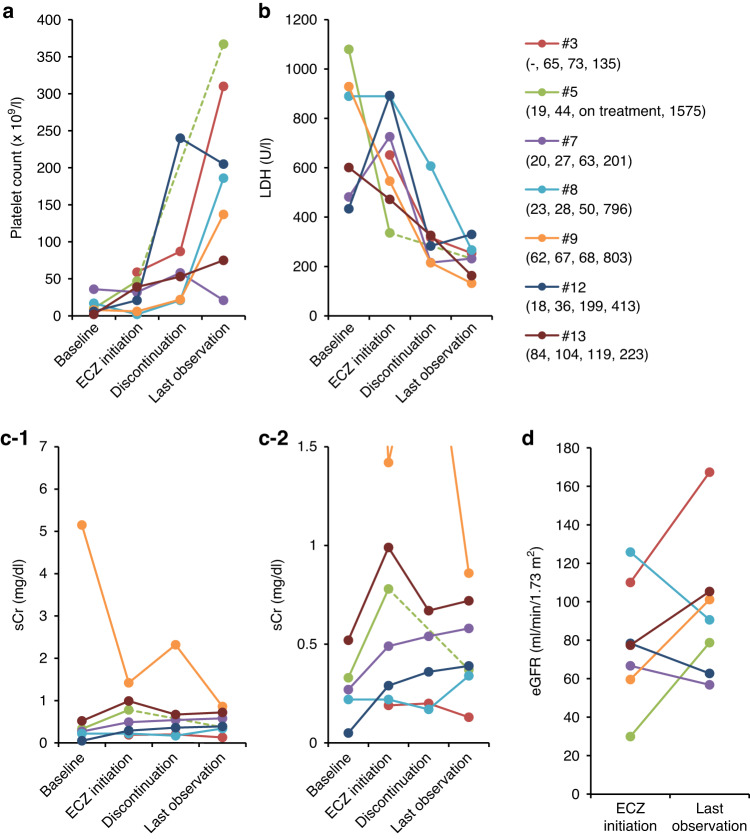
Changes in laboratory parameters over time in surviving patients. **a** Platelet count, **b** lactate dehydrogenase, **c-1**, **c-2** serum creatinine, and **d** estimated glomerular filtration rate. Values are shown for individual patients. Days after HSCT are shown under each patient number (from HSCT to baseline, to ECZ initiation, to discontinuation, and to last observation, respectively).

LDH levels significantly decreased during eculizumab treatment (median 22 days, IQR 11.5–99.5 days) and platelet counts significantly increased from eculizumab initiation to the last observation period (median 280 days, IQR 124–730.5 days) (Tables 4, S5, Fig. 3). Even though the median sCr did not change during the observation period, 4 out of 7 survivors showed sCr improvement and all three patients who were on dialysis at the initiation of eculizumab discontinued dialysis by the last observation (Table S5). None of the survivors experienced recurrence of TMA during the subsequent follow-up period (median 111.5 days, IQR 95–555; calculated from the last dose of eculizumab to the end of observation in six surviving patients who discontinued eculizumab).

**Table 4:** Changes in laboratory parameters in surviving patients (*n* = 7).

Laboratory parameter	At initiation of ECZ		At discontinuation of ECZ		*P*^a^	Last observation		*P*^a^
PLT count, ×10^9^/L	32	(14–43)	58	(38–164)	0.073	186	(106–258)	0.011
LDH, U/L	652	(509-808)	283	(224–322)	<0.01	232	(198–261)	<0.01
sCr, mg/dL	0.49	(0.26-0.89)	0.37	(0.28–0.61)	0.79	0.39	(0.36–0.65)	0.79

Values are median (IQR).

*ECZ* eculizumab, *eGFR* estimated glomerular filtration rate, *LDH* lactate dehydrogenase, *PLT* platelet, *sCr* serum creatinine.

^a^*P*-values versus at the initiation of eculizumab.

### Non-survivors

**Fig. 4:**
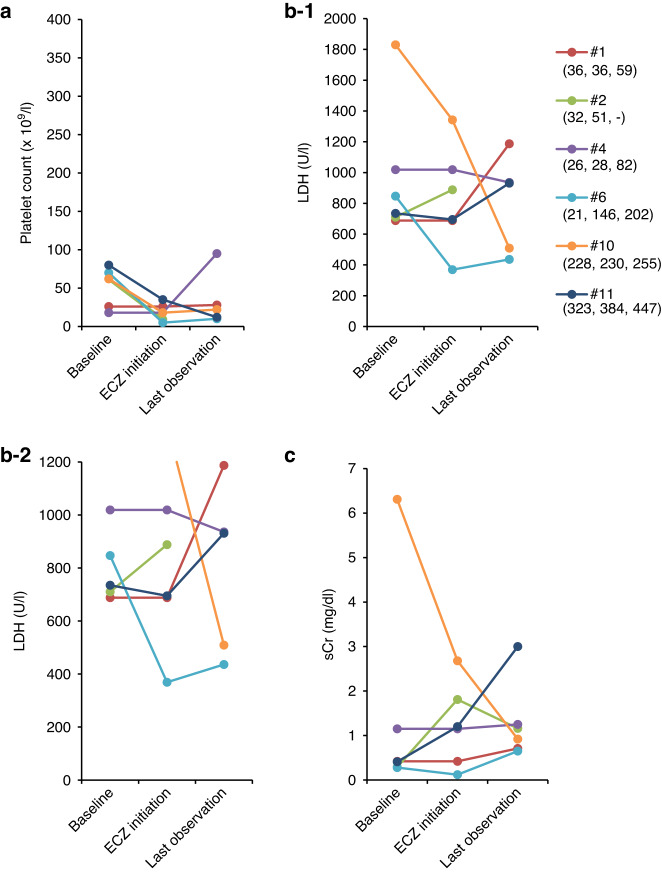
Changes in laboratory parameters over time in deceased patients. **a** platelet count, **b-1**, **b-2** lactate dehydrogenase, and **c** serum creatinine. Values are shown for individual patients. Days after HSCT are shown under each patient number (from HSCT to baseline, to ECZ initiation, and to last observation, respectively).

The details of the 6 non-survivors are described in Supplemental Table 4 and Fig. 4. In summary, three patients (#1, #4, #10) died due to the ongoing adverse events initiated prior to first eculizumab dose, two patients (#2, #6) died due to adverse events caused by infections; both patients were treated with immunosuppressive therapy to manage ongoing GVHD, and one patient (#11) did not show platelet count recovery during eculizumab treatment and died of TMA. No improvement trend was observed in any of the parameters measured.

### Comparison between survivors and non-survivors

No significant differences were observed in the period from HSCT to TMA onset and the period from TMA onset to eculizumab initiation between survivors and non-survivors (Table S6). The number of patients who received 4 or more doses of eculizumab was similar between survivors and non-survivors (3/7 and 3/6 respectively). Although platelet count, LDH and serum creatinine levels were not different at the initiation of eculizumab, they showed a significant difference at the last observation (Fig. S1 and Table S6).

## Discussion

This study is a real-world analysis of 13 paediatric patients with TMA after HSCT who were treated with eculizumab in accordance with the approved regimen for aHUS. Eculizumab treatment appeared to show benefit in over half of these patients with favourable haematologic responses observed after the initiation of eculizumab in survivors. Three of seven survivors received less than 4 doses of eculizumab, and no TMA recurrence was observed until the last observation.

All 13 patients analysed here were clinically diagnosed with aHUS according to diagnostic criteria in the latest Japanese aHUS clinical guides at the time of diagnosis. According to the clinical guide, patients might be diagnosed with aHUS, even if they did not display all symptoms of aHUS. Accordingly, two patients analysed here did not have acute kidney injury (AKI) at TMA onset; one patient experienced exacerbation of renal function 17 days after TMA onset and the other had extra-renal organ damage caused by TMA. In addition to the items of the aHUS diagnostic criteria, the proposed HSCT-TMA diagnostic criteria include parameters of hypertension, proteinuria, and elevated sC5b-9 [9]. Twelve out of thirteen patients experienced hypertension and/or proteinuria. Although sC5b-9 level data was not collected, all patients fulfilled ≥4 of items of the algorithm, satisfying the HSCT-TMA diagnostic criteria as defined by Schoettler, M. L., et al.

In this study, 54% (7/13) of patients survived to the end of the observation period (median 172 days, IQR 66–396). This survival rate was similar to results obtained in a recent meta-analysis; 52% (95CI: 40–65%) of patients treated with eculizumab survived [5]. In the report by Jodele, et al., the survival rate of patients treated with eculizumab was as high as 77% at 6 months from HSCT-TMA diagnosis. Jodele’s cohort consisted of patients with high-risk HSCT-TMA (hrHSCT-TMA) as defined by high complement complex activity (sC5b-9) level and the existence of proteinuria [32]. The patients included in the cohort were treated with eculizumab as first line therapy and received intensive treatment with personalized dosing different from the aHUS label as determined by assessments of CH50 level and blood concentration of eculizumab [32]. In eculizumab PMS, CH50 level and blood concentration data were not collected, and all patients analysed here did not receive an intensive personalized treatment; therefore, we could not assess if eculizumab dosing was adequate to suppress complement activity. This difference could explain the better survival outcomes overserved in the Jodele’s cohort.

Renal function improvement has been observed in patients with aHUS who initiated eculizumab treatment early [33]. In patients with HSCT-TMA, early diagnosis and appropriate treatment intervention are also important for better prognosis. The delay of eculizumab initiation has been proposed to lead to death due to multi-organ dysfunction [32], and plasma therapy in HSCT-TMA has resulted in a low response rate and high mortality [3]. In the current analysis, 8 out of 13 patients received plasma therapy before eculizumab treatment for clinically diagnosed aHUS, while eculizumab was used as a first-line therapy for HSCT-TMA in the report from Jodele, et al., which achieved a higher survival rate [32]. While not statistically significant, both the period from HSCT to TMA onset and the period from TMA onset to eculizumab administration were numerically shorter in survivors (21 days vs. 31.5 days and 8 days vs. 18 days, respectively) in this analysis. Early treatment with complement inhibitor and early evaluation of treatment response can be a preferential treatment strategy once complement dysfunction is suspected in HSCT-TMA.

In previous PMS reports, paediatric patients with aHUS showed rapid improvement in both platelet count and LDH levels after eculizumab initiation, while they showed delayed improvement in renal function [25, 26]. In the subpopulation analysed here, the seven surviving patients with HSCT-TMA did not show evidence of platelet count recovery during the first 22 days (median, IQR: 11.5–99.5) of eculizumab treatment. However, improvements were observed at the last observation; 280 days after eculizumab initiation (median, IQR 124–730.5 days). This delay of platelet response might have been due to insufficient haematopoietic recovery after HSCT. Renal improvement was not observed in three survivors; there is a possibility that the treatment period or blood concentration of eculizumab might not be enough to observe an improvement in renal function; otherwise, renal damage in those patients was already irreversible. Taken together, LDH levels may be a better marker to evaluate an early response to eculizumab in patients with HSCT-TMA.

In a previous report from Jodele et al., no difference in baseline laboratory parameters (platelet count, LDH, and sCr level) was identified between survivors and non-survivors [13]. Similarly, in our analysis, no significant difference was observed in the baseline laboratory parameters suggesting these parameters do not provide insights on patients’ outcomes. In addition, the ratio of any of complications (GVHD, VOD or infectious disease) and the existence of complement gene variant were also similar between the survivors and non-survivors, which was consistent with previous studies [16, 32]. Although the levels of proteinuria and sC5b-9 at baseline have been suggested as potential risk factors in patients developing HSCT-TMA [13, 32], we could not evaluate sC5b-9 level in the PMS because it was not collected. In a previous meta-analysis, GVHD, infection, and TMA related organ failure were the main causes of death in patients with HSCT-TMA who were treated with eculizumab (estimated proportions are 26%, 31 and 23 %, respectively) [5]. These factors were also the main cause of death in a previously reported paediatric population [32]. In our current analysis, one or more of these factors were observed in all six non-survivors. These results reflect the complexity of complications after HSCT, and suggest that future studies should consider the management of these complications during complement C5 inhibitor treatment for HSCT-TMA.

The small number of patients and broad variability of patient baseline characteristics prevented us from making robust analyses to identify possible risk factors for HSCT-TMA outcomes. In addition, only patients clinically diagnosed with aHUS by their treating physician after HSCT were analysed here; in other words, patients with HSCT-TMA who did not receive a diagnosis of aHUS were excluded from this analysis. These limitations should be carefully considered when interpreting and assessing the generalizability of our findings.

In conclusion, results from our analyses showed that administration of eculizumab resulted in clinically relevant haematological responses and favourable outcomes in a cohort of paediatric patients with HSCT-TMA. Ongoing clinical studies are expected to elucidate the optimized intensive treatment regimen of the long-acting terminal complement inhibitor, ravulizumab, specifically for HSCT-TMA [34, 35] to further improve the treatment outcome. Also, further research into the risk stratification of HSCT-TMA and the use of C5 inhibitors are needed to confirm appropriate use in HSCT-TMA and to identify factors that might predict patients’ responses to therapy.

### Supplementary information

Supplemental materials

## Data Availability

The data underlying this article are available in the article and in its online supplementary data.

## References

[1] Gavriilaki E, Sakellari I, Anagnostopoulos A, Brodsky RA (2017). Transplant-associated thrombotic microangiopathy: opening Pandora’s box. Bone Marrow Transpl.

[2] Hematopoietic Cell Transplantation in Japan Annual Report of Nationwide Survey 2021. http://www.jdchct.or.jp/ Accessed September 28, 2022.

[3] Ho VT, Cutler C, Carter S, Martin P, Adams R, Horowitz M (2005). Blood and marrow transplant clinical trials network toxicity committee consensus summary: thrombotic microangiopathy after hematopoietic stem cell transplantation. Biol Blood Marrow Transpl.

[4] Elsallabi O, Bhatt VR, Dhakal P, Foster KW, Tendulkar KK (2016). Hematopoietic stem cell transplant-associated thrombotic microangiopathy. Clin Appl Thromb Hemost.

[5] Zhang R, Zhou M, Qi J, Miao W, Zhang Z, Wu D (2021). Efficacy and Safety of Eculizumab in the Treatment of Transplant-Associated Thrombotic Microangiopathy: A Systematic Review and Meta-Analysis. Front Immunol.

[6] Jodele S, Dandoy CE, Myers KC, El-Bietar J, Nelson A, Wallace G (2016). New approaches in the diagnosis, pathophysiology, and treatment of pediatric hematopoietic stem cell transplantation-associated thrombotic microangiopathy. Transfus Apher Sci.

[7] Jodele S, Sabulski A (2021). Transplant-associated thrombotic microangiopathy: elucidating prevention strategies and identifying high-risk patients. Expert Rev Hematol.

[8] Shen YM (2016). Clinical evaluation of thrombotic microangiopathy: identification of patients with suspected atypical hemolytic uremic syndrome. Thromb J.

[9] Schoettler ML, Carreras E, Cho B, Dandoy CE, Ho VT, Jodele S (2023). Harmonizing Definitions for Diagnostic Criteria and Prognostic Assessment of Transplantation-Associated Thrombotic Microangiopathy: A Report on Behalf of the European Society for Blood and Marrow Transplantation, American Society for Transplantation and Cellular Therapy, Asia-Pacific Blood and Marrow Transplantation Group, and Center for International Blood and Marrow Transplant Research. Transplant Cell Ther.

[10] Ruutu T, Barosi G, Benjamin RJ, Clark RE, George JN, Gratwohl A (2007). Diagnostic criteria for hematopoietic stem cell transplant-associated microangiopathy: results of a consensus process by an International Working Group. Haematologica.

[11] Young JA, Pallas CR, Knovich MA (2021). Transplant-associated thrombotic microangiopathy: theoretical considerations and a practical approach to an unrefined diagnosis. Bone Marrow Transpl.

[12] Cho BS, Yahng SA, Lee SE, Eom KS, Kim YJ, Kim HJ (2010). Validation of recently proposed consensus criteria for thrombotic microangiopathy after allogeneic hematopoietic stem-cell transplantation. Transplantation.

[13] Jodele S, Davies SM, Lane A, Khoury J, Dandoy C, Goebel J, et al. Diagnostic and risk criteria for HSCT-associated thrombotic microangiopathy: a study in children and young adults. Blood. 2014;124:645–53. 10.1182/blood-2014-03-564997 PMC4110664 24876561

[14] Goodship TH, Cook HT, Fakhouri F, Fervenza FC, Frémeaux-Bacchi V, Kavanagh D (2017). Atypical hemolytic uremic syndrome and C3 glomerulopathy: conclusions from a “Kidney Disease: Improving Global Outcomes” (KDIGO) controversies conference. Kidney Int.

[15] Okamura H, Nakamae H, Shindo T, Ohtani K, Hidaka Y, Ohtsuka Y (2021). Early elevation of complement factor Ba is a predictive biomarker for transplant-associated thrombotic microangiopathy. Front Immunol.

[16] Jodele S, Zhang K, Zou F, Laskin B, Dandoy CE, Myers KC, et al. The genetic fingerprint of susceptibility for transplant-associated thrombotic microangiopathy. Blood. 2016;127:989–96. 10.1182/blood-2015-08-663435 PMC4828073 26603840

[17] Rosenthal J (2016). Hematopoietic cell transplantation-associated thrombotic microangiopathy: a review of pathophysiology, diagnosis, and treatment. J Blood Med.

[18] Seaby EG, Gilbert RD (2018). Thrombotic microangiopathy following haematopoietic stem cell transplant. Pediatr Nephrol.

[19] Genere L, Bacchetta J, Bertrand Y, Javouhey E, Cheikh N, Sellier-Leclerc AL (2018). Eculizumab and thrombotic microangiopathy after hematopoietic stem cell transplantation: a report on its efficacy and safety in two pediatric patients. Arch Pediatr.

[20] Jodele S, Fukuda T, Vinks A, Mizuno K, Laskin BL, Goebel J (2014). Eculizumab therapy in children with severe hematopoietic stem cell transplantation-associated thrombotic microangiopathy. Biol Blood Marrow Transpl.

[21] Schoettler M, Lehmann L, Li A, Ma C, Duncan C (2019). Thrombotic microangiopathy following pediatric autologous hematopoietic cell transplantation: a report of significant end-organ dysfunction in eculizumab-treated survivors. Biol Blood Marrow Transpl.

[22] Epperla N, Hemauer K, Hamadani M, Friedman KD, Kreuziger LB (2017). Impact of treatment and outcomes for patients with posttransplant drug-associated thrombotic microangiopathy. Transfusion.

[23] Schoettler M, Duncan C, Lehmann L (2019). Severe, persistent neurotoxicity after transplant-associated thrombotic microangiopathy in a pediatric patient despite treatment with eculizumab. Pediatr Transpl.

[24] SOLIRIS® (eculizumab) 300 mg for intravenous infusion. Interview Form, revised December 2020 (65th edition). https://soliris.jp/-/media/soliris_jp/document-slide/interview_form.pdf. Accessed December 3, 2020.

[25] Ito N, Hataya H, Saida K, Amano Y, Hidaka Y, Motoyoshi Y (2016). Efficacy and safety of eculizumab in childhood atypical hemolytic uremic syndrome in Japan. Clin Exp Nephrol.

[26] Ito S, Hidaka Y, Inoue N, Kaname S, Kato H, Matsumoto M (2019). Safety and effectiveness of eculizumab for pediatric patients with atypical hemolytic-uremic syndrome in Japan: interim analysis of post-marketing surveillance. Clin Exp Nephrol.

[27] Ito S, Hataya H, Ashida A, Hamada R, Ishikawa T, Ishikawa Y, et al. Eculizumab for paediatric patients with atypical haemolytic-uremic syndrome: Full dataset analysis of post-marketing surveillance in Japan. Nephrol Dial Transplant, 2022. 10.1093/ndt/gfac150. 10.1093/ndt/gfac150 PMC9923705 35438790

[28] Sawai T, Nangaku M, Ashida A, Fujimaru R, Hataya H, Hidaka Y (2014). Diagnostic criteria for atypical hemolytic uremic syndrome proposed by the Joint Committee of the Japanese Society of Nephrology and the Japan Pediatric Society. Clin Exp Nephrol.

[29] Kato H, Nangaku M, Hataya H, Sawai T, Ashida A, Fujimaru R (2016). Clinical guides for atypical hemolytic uremic syndrome in Japan. Clin Exp Nephrol.

[30] Kato H, Nangaku M, Okada H, Kagami S (2018). Controversies of the classification of TMA and the terminology of aHUS. Clin Exp Nephrol.

[31] Uemura O, Nagai T, Ishikura K, Ito S, Hataya H, Gotoh Y (2014). Creatinine-based equations to estimate glomerular filtration rate in Japanese children and adolescents with chronic kidney disease. Clin Exp Nephrol.

[32] Jodele S, Dandoy CE, Lane A, Laskin BL, Teusink-Cross A, Myers KC, et al. Complement blockade for TA-TMA: lessons learned from a large pediatric cohort treated with eculizumab. Blood. 2020;135:1049–57. 10.1182/blood.2019004218 PMC7099329 31932840

[33] Walle JV, Delmas Y, Ardissino G, Wang J, Kincaid JF, Haller H (2017). Improved renal recovery in patients with atypical hemolytic uremic syndrome following rapid initiation of eculizumab treatment. J Nephrol.

[34] Ravulizumab in Thrombotic Microangiopathy After Hematopoietic Stem Cell Transplant. https://clinicaltrials.gov/ct2/show/NCT04543591 Accessed August 5, 2022

[35] Study of Ravulizumab in Pediatric Participants With HSCT-TMA. https://clinicaltrials.gov/ct2/show/NCT04557735 Accessed August 5, 2022

[36] Hasegawa D, Saito A, Nino N, Uemura S, Takafuji S, Yokoi T (2018). Successful Treatment of Transplantation-associated Atypical Hemolytic Uremic Syndrome With Eculizumab. J Pediatr Hematol Oncol.

[37] Nozawa A, Ozeki M, Hori T, Kawamoto N, Hirayama M, Azuma E (2018). A Heterozygous CFHR3-CFHR1 Gene Deletion in a Pediatric Patient With Transplant-associated Thrombotic Microangiopathy Who was Treated With Eculizumab. J Pediatr Hematol Oncol.

[38] Yamada A, Moritake H, Kinoshita M (2017). Prompt Improvement of Transplant-associated Thrombotic Microangiopathy by Eculizumab Administration for Neuroblastoma after Autologous Hematopoietic Stem Cell Transplantation. J Jpn Pediatr Soc.

[39] Shimizu S, Morohashi T, Kanezawa K, Yagasaki H, Takahashi S, Morioka I. Case Report: Successful Treatment With Anti-C5 Monoclonal Antibody in a Japanese Adolescent Who Developed Thrombotic Microangiopathy After Autologous Bone Marrow Transplantation for Malignant Lymphoma. Front Pediat. 2022;10:908183. 10.3389/fped.2022.908183 PMC9289264 35859949

