# Supplementary material for: Eculizumab treatment in paediatric patients diagnosed with aHUS after haematopoietic stem cell transplantation: a HSCT-TMA case series from Japanese aHUS post-marketing surveillance
Source: Bone Marrow Transplant. 2023 Dec 15;59(3):315–24. doi: 10.1038/s41409-023-02161-7 (PMC10920193; doi:10.1038/s41409-023-02161-7)
Supplement: Supplementary file 1 — Supplemental materials [file 41409_2023_2161_MOESM1_ESM.pdf]

Table S1. Items for the aHUS and HSCT-TMA diagnostic algorithms and the status of the patients at aHUS diagnosis

Pt No	TMA triad			Extra-renal damage	Schistocytes	TTP (ADAMTS 13 activity <10%)	STEC-HUS	Items included in HSCT-TMA diagnosis algorithms (Schoettler, *et al*. [9])		HSCT-TMA diagnosis algorithms [9][d]
	Thrombocytopenia	MAHA	AKI					Hypertension	Proteinuria	
1*	Yes	Yes	No[a]	Pulmonary	n/a	Excluded	Excluded	Detected	n/a	Met
2*	Yes	Yes	Yes	Nervous, Gastrointestinal	Detected	Excluded	n/a	Detected	n/a	Met
3	Yes	Yes	No	Pulmonary [b]	Detected	Excluded	n/a [c]	Detected	n/a	Met
4*	Yes	Yes	Yes	Gastrointestinal	Detected	n/a	n/a [c]	Detected	n/a	Met
5	Yes	Yes	Yes	Nervous [b], Gastrointestinal [b]	Detected	Excluded	Excluded	Detected	Detected	Met
6*	Yes	Yes	Yes	Pulmonary, Gastrointestinal	n/a	Excluded	Excluded	Detected	Detected	Met
7	Yes	Yes	Yes	Pulmonary [b]	n/a	Excluded	n/a [c]	Detected	Detected	Met
8	Yes	Yes	Yes	Pulmonary [b], Eye [b]	Detected	Excluded	Excluded	Detected	Detected	Met
9	Yes	Yes	Yes	Gastrointestinal [b]	n/a	Excluded	Excluded	n/a	n/a	Met
10*	Yes	Yes	Yes	Pulmonary, Nervous, Liver	Detected	Excluded	Excluded	n/a	Detected	Met
11*	Yes	Yes	Yes	No	n/a	Excluded	Excluded	Detected	n/a	Met
12	Yes	Yes	Yes	No	n/a	Excluded	Excluded	Detected	Detected	Met
13	Yes	Yes	Yes	Pulmonary [b]	n/a	Excluded	n/a [c]	Detected	Detected	Met

*non-survivors
[a] Exacerbation of renal function after 17 days from TMA onset
[b] Resolved after eculizumab administration
[c] No diarrhea nor bloody stools
[d] Must meet ≥4 of following items: thrombocytopenia, anemia, elevated LDH, schistocytes, hypertension, elevated sC5b-9, and proteinuria
aHUS, atypical haemolytic–uremic syndrome; TMA, thrombotic microangiopathy; MAHA, microangiopathic haemolytic anemia; AKI, acute kidney injury; LDH, lactate dehydrogenase; n/a, not available; PLT, platelet; TTP, thrombotic thrombocytopenic purpura; STEC, Shiga toxin–producing *Escherichia coli*

Table S2. Treatment for TMA before eculizumab administration

Pt No.	PE/PI	Dialysis	Steroids	Immunosuppressive drugs	Other drugs	Platelet transfusion	Renal transplantation
1*	PE/PI	-	-	-	NM	-	-
2*	PE	Done	-	-	-	Done	-
3	PI	-	-	-	-	Done	-
4*	-	Done	Done	-	-	Done	-
5	PE/PI	Done	Done	-	-	Done	-
6*	-	-	-	-	rTM, PHL, ADB	Done	-
7	PI	-	-	-	-	-	-
8	PI	-	-	-	-	Done	-
9	PE/PI	Done	-	-	-	-	-
10*	-	Done	-	-	-	Done	-
11*	PI	-	-	-	rTM	-	-
12	-	-	Done	-	rTM	Done	-
13	PE/PI	Done	-	-	-	Done	-

*non-survivors
NM, nafamostat mesilate; rTM, recombinant thrombomodulin; PHL, propranolol hydrochloride; ADB, amlodipine besilate

Table S3. All medicines used in the past 1 year before aHUS diagnosis

Pt No.	Drugs
1*	Nicardipine Hydrochloride, Carperitide, Hydrocortisone Sodium Succinate, rTM, Mirimostim, Lenograstim, Filgrastim, Nafamostat Mesilate, Tacrolimus Hydrate, Cyclophosphamide Hydrate, Melphalan, Methotrexate, Fludarabine Phosphate, Etoposide, Carboplatin, Teicoplanin, Teicoplanin, Cefepime Dihydrochloride Hydrate, Tazobactam Piperacillin Hydrate, Amphotericin B, Voriconazole, Levofloxacin Hydrate, Ganciclovir, anti-thrombin III
2*	Midazolam, Furosemide, Omeprazole, Prednisolone, Octreotide Acetate, Monoammonium Glycyrrhizinate, Glutathione, Tacrolimus Hydrate, Busulfan, Melphalan, Methotrexate, Fludarabine Phosphate, Vancomycin Hydrochloride, Micafungin Sodium, Aciclovir, anti-thrombin III, Anti-human Thymocyte Immunoglobulin, Fentanyl Citrate
3	Dalteparin sodium, rTM, Cyclophosphamide Hydrate, Busulfan, Methotrexate, Cytarabine, Daunorubicin Hydrochloride, Doxorubicin Hydrochloride, Vincristine Sulfate, Etoposide, L-Asparaginase, Haptoglobin
4*	Furosemide, Tacrolimus Hydrate, Cyclophosphamide Hydrate, Ifosfamide, Busulfan, Melphalan, Fludarabine Phosphate, Pirarubicin, Vincristine Sulfate, Etoposide, Cisplatin
5	Prednisolone, rTM, Melphalan, Fludarabine Phosphate, Etoposide
6*	Propranolol Hydrochloride, Amlodipine Besilate, Sildenafil Citrate, Lansoprazole, Ursodeoxycholic Acid, Prednisolone, rTM, beraprost, Beraprost Sodium, Mycophenolate Mofetil, Tacrolimus Hydrate, Melphalan, Fludarabine Phosphate, Aciclovir, Ganciclovir, Sulfamethoxazole Trimethoprim
7	Nicardipine Hydrochloride, Cyclophosphamide Hydrate, Melphalan, Pirarubicin, Vincristine Sulfate, Etoposide, Cisplatin, Carboplatin
8	Cilnidipine, Hydrocortisone, Fludrocortisone Acetate, Hydrocortisone Sodium Succinate, Prednisolone, Dalteparin sodium, Lenograstim, Melphalan, Etoposide, Carboplatin, Teicoplanin, Cefozopran Hydrochloride, Meropenem Hydrate, Aciclovir, Immunoglobulin
9	Olprinone Hydrochloride Hydrate, Prednisolone, rTM, Filgrastim, Ciclosporin, Cyclophosphamide Hydrate, Melphalan, Clofarabine, Daunorubicin Hydrochloride, Vincristine Sulfate, Etoposide, L-Asparaginase, Rituximab, Meropenem Hydrate
10*	Ursodeoxycholic Acid, Prednisolone, Mycophenolate Mofetil, Tacrolimus Hydrate, Ifosfamide, 6-Mercaptopurine Riboside, Methotrexate, Cytarabine, Nelarabine, Daunorubicin Hydrochloride, Etoposide, Amphotericin B, Fluconazole
11*	rTM, Mycophenolate Mofetil, Foscarnet Sodium Hydrate, Brotizolam, Furosemide, Doxazosin Mesilate, Olmesartan Medoxomil, Nifedipine, Famotidine, Prednisolone
12	rTM, Tacrolimus Hydrate, Melphalan, Methotrexate, Fludarabine Phosphate
13	Prednisolone, Cyclophosphamide Hydrate, Ifosfamide, Melphalan, Methotrexate, Cytarabine, Fludarabine Phosphate, Pirarubicin, Vincristine Sulfate, Etoposide, Carboplatin, Rituximab

*non-survivors

Table S4. Adverse events during eculizumab treatment

Pt No.	SAE during ECZ treatment	Details of SAE leading to death
1*	Multiple organ dysfunction syndrome[a]	The patient had ongoing pulmonary haemorrhage at the onset of TMA and died after 29 days due to the worsening multiple organ dysfunction syndrome. Eculizumab treatment was continued until the time of death. The multiple organ dysfunction syndrome was not related to eculizumab. The relationships between ongoing primary disease (neuroblastoma) and death were not recorded.
2*	Bacteraemia[b], Asphyxia, White blood cell count decreased[b], Adenovirus infection[a]	The patient was treated with immunosuppressive drugs for post-transplant GVHD. The patient got infected with adenovirus during eculizumab treatment and discontinued its treatment. Due to the complications of adenovirus infection and TMA, the patient died. These complications were not related to eculizumab. The relationships between ongoing primary disease (AML) and death were not recorded.
3	None	-
4*	Pyrexia, Cerebral haemorrhage[a]	The patient had ongoing hypertension since the onset of TMA and died after 57 days due to cerebral haemorrhage caused by the hypertension. Eculizumab treatment was continued until the time of death. The cerebral haemorrhage was not related to eculizumab. The relationships between ongoing primary disease (neuroblastoma) and death were not recorded.
5	Platelet count decreased, Infection, Pulmonary hypertension, Respiratory tract infection	-
6*	Pseudomonas sepsis[a]	The patient was treated with immunosuppressive drugs for post-transplant GVHD. The patient experienced pseudomonas sepsis during the treatment and died the next day. Eculizumab treatment was continued until the time of death. The pseudomonas sepsis was not related to eculizumab. The relationships between ongoing primary disease (FHL) and death were not recorded.
7	None	-
8	Pulmonary haemorrhage	-
9	Candida sepsis, Bronchopulmonary aspergillosis, Organising pneumonia	-
10*	Pulmonary alveolar haemorrhage[a]	The patient had invasive bronchopulmonary aspergillosis before eculizumab treatment. The patient died due to pulmonary alveolar haemorrhage caused by invasive bronchopulmonary aspergillosis. Eculizumab treatment was continued until the time of death. The pulmonary alveolar haemorrhage was not related to eculizumab. Relapse of the primary disease (ALL) was not recorded after HSCT and did not related to the death.
11*	TMA[a]	No platelet count increasement was seen after five doses of eculizumab. The patient died due to TMA. Eculizumab treatment was continued until the time of death. The TMA was not related to eculizumab. Relapse of the primary disease (ALL) was not recorded after HSCT and did not related to the death.
12	None	-
13	None	-

*non-survivors
[a]The causes of death recorded as adverse events
[b]Drug related serious adverse event
SAE, serious adverse event; CFH, complement factor H; ECZ, eculizumab; LDH, lactate dehydrogenase; PLT, platelet; Pt, patient; TMA, thrombotic microangiopathy; WBC, white blood cell

Table S5. Outcomes at the last observation (survivors)

Pt No	At ECZ initiation					At ECZ discontinuation				Time (days)		At last observation				
	PLT count ($\times 10^9$/l)	LDH (U/l)	sCr (mg/dl)	eGFR (ml/min/ 1.73 m^2)	Dialysis /transfusion	PLT count	LDH	sCr	Dialysis /transfusion	ECZ duration	ECZ discontinuation to last observation	PLT count	LDH	sCr	eGFR	Dialysis/ transfusion
3	59	652[††]	0.19	110.1	No/Yes	87	317	0.2	No/No	8	47	310	254	0.13	167.4	No/No
5	47	336	0.78	29.9	Yes/Yes	-	-	-	-	1342	ECZ ongoing	367	232	0.37	78.8	No/No
7	32	726[††]	0.49	66.7	No/Yes	58	216	0.54	No/Yes	36	91	21	232	0.58	56.8	No/No
8	2	890[†††]	0.22	125.8	No/Yes	21	607[††]	0.17	No/Yes	22	701	186	267	0.34	90.6	No/No
9	6	546[†††]	1.42	59.6	Yes/Yes	22	215	2.32	Yes/Yes	1	737	137	132	0.86	101.1	No/No
12	21	893[†††]	0.29	78.4	No/Yes	240	283	0.36	No/No	163	117	205	330	0.39	62.7	No/No
13	39	472[††]	0.99	77.4	Yes/No	53	326[†]	0.67	No/No	15	106	75	163	0.72	105.4	No/No

ECZ, eculizumab; eGFR, estimated glomerular filtration rate; LDH, lactate dehydrogenase; PLT, platelet; Pt, patient; sCr, serum creatinine
[†††]> 2x upper limit of normal (ULN) of LDH, [††]> 1.5x ULN , [†]> 1x ULN of the patient age.

Table S6. Comparison between patients who survived and died.

	**Surviving**	**Deceased**	***p***
**From HSCT to TMA onset (days)**	21 (19.5-55)	31.5 (27.3-169.3)	0.22
**From TMA onset to ECZ initiation (days)**	8 (8-22.5)	18 (8.5-51.5)	0.72
**From TMA onset to the last observation (days)**	396 (177-759)	45 (61.5-110.25)	0.012
**No. of ECZ doses (times)**	3 (2.5-7)	3.5 (2.3-4.8)	0.94
**PLT count at initiation of ECZ, $\times 10^9$/l**	32 (14-43)	22 (11-33)	0.83
**PLT count at the last observation, $\times 10^9$/l**	186 (106-258)	22 (12-28)	0.03
**LDH at initiation of ECZ, U/l**	652 (509-808)	792 (690-986)	0.35
**LDH at last observation, U/l**	232 (198-261)	931 (509-936)	<0.01
**sCr at initiation of ECZ, mg/dl**	0.49 (0.26-0.89)	1.18 (0.6-1.66)	0.28
**sCr at last observation, mg/dl**	0.39 (0.36-0.65)	1.04 (0.76-1.23)	0.014

Values are median (IQR)
ECZ, eculizumab; LDH, lactate dehydrogenase; PLT, platelet

Supplemental Figure 1. Baseline levels of thrombotic microangiopathy-associated laboratory parameters in surviving and deceased patients.

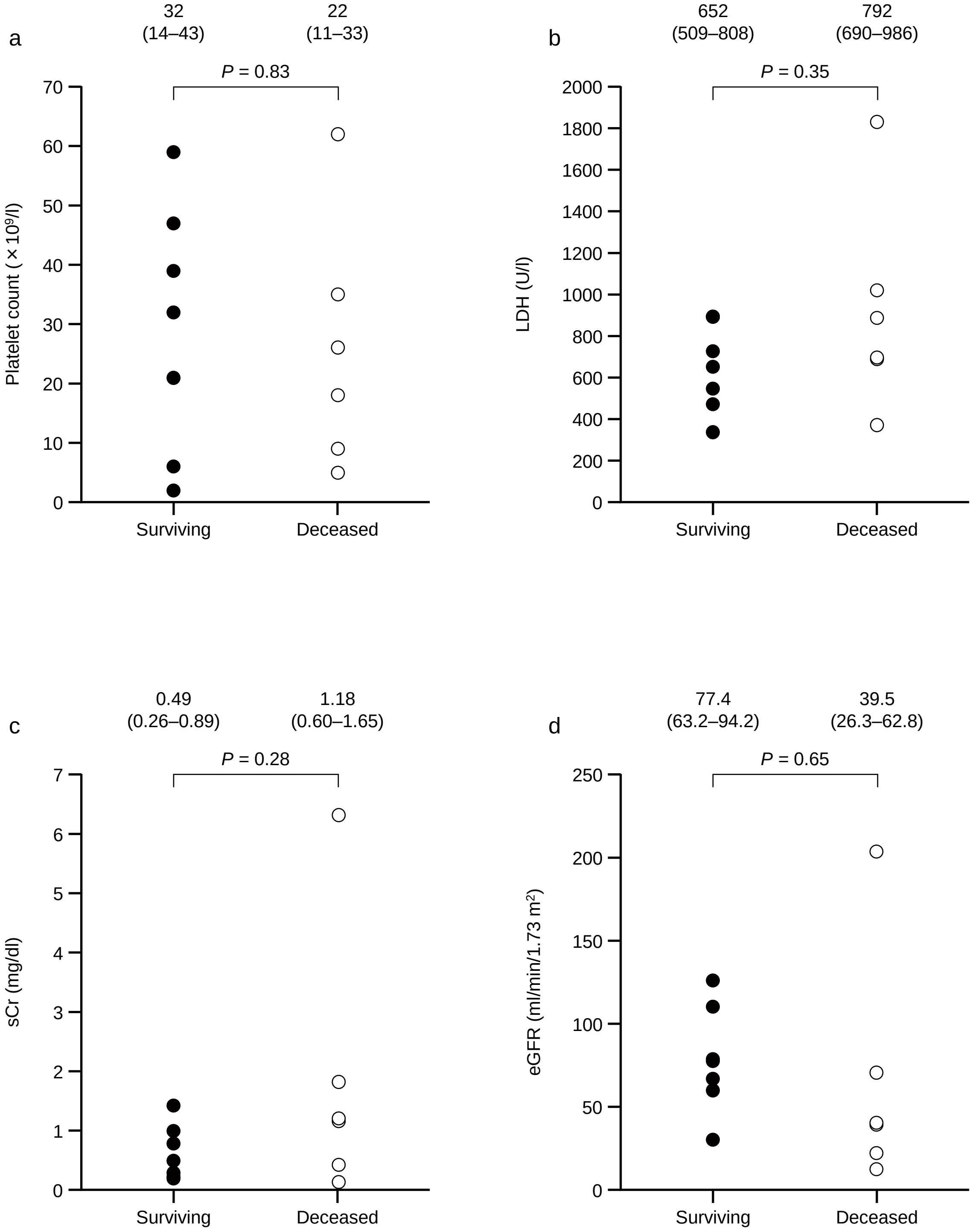

(a) platelet count, (b) lactate dehydrogenase, (c) serum creatinine, and (d) estimated glomerular filtration rate
Values are shown for individual patients, with the median (IQR) above each graph.
P-values were determined using Wilcoxon's rank sum test.
Estimated glomerular filtration rate (eGFR) was calculated using the following formulas specified for Japanese patients aged 2 years through 18 years, with "X" as body height (m) : eGFR = 110.2*[- 1.259X^5 + 7.815X^4 – 18.57X^3 + 21.39X^2 – 11.71X + 2.628]/(serum creatinine, sCr) + 2.93 for boys and 110.2*[- 4.536 X^5 + 27.16 X^4 – 63.47 X^3 + 72.43 X^2 – 40.06X + 8.778]/(sCr) + 2.93 for girls [31].
